# Both Ser361 phosphorylation and the C‐arrestin domain of thioredoxin interacting protein are important for cell cycle blockade at the G1/S checkpoint

**DOI:** 10.1002/2211-5463.12518

**Published:** 2018-10-01

**Authors:** Kazuyo Kamitori, Fuminori Yamaguchi, Youyi Dong, Akram Hossain, Ayako Katagi, Chisato Noguchi, Yuko Hirata, Ikuko Tsukamoto, Naoya Hatano, Masaaki Tokuda

**Affiliations:** ^1^ Departments of Cell Physiology Faculty of Medicine Kagawa University Miki‐cho Japan; ^2^ Department of Pharmaco‐Bio‐Informatics Faculty of Medicine Kagawa University Miki‐cho Japan; ^3^ Integrated Center for Mass Spectrometry Kobe University Graduate School of Medicine Japan

**Keywords:** anti‐tumor, arrestin, cell cycle, p27^kip1^, p38 MAPK, TXNIP

## Abstract

Thioredoxin interacting protein (TXNIP) is a novel tumor suppressor that is down‐regulated in several cancer tissues and tumor cell lines. Overexpression of TXNIP causes cell cycle arrest at the G1/S checkpoint in the hepatocellular carcinoma cell line HuH‐7. TXNIP contains putative phosphorylation sites, but the effects of its phosphorylation have not been fully characterized. TXNIP also contains two α‐arrestin domains (N‐arrestin and C‐arrestin) whose functions are not fully understood. Here, we reveal an association between TXNIP and cell cycle regulatory proteins (p27^kip1^, Jun activation domain‐binding protein 1 (JAB1), Cdk2, and cyclin E), suggesting its participation in cell cycle regulation. We observed phosphorylation of TXNIP and used both *in vivo* and *in vitro* kinase assays to demonstrate that TXNIP can be phosphorylated by p38 mitogen‐activated protein kinase. Furthermore, we also identified Ser361 in TXNIP as one of the major phosphorylation sites. Cell cycle analysis showed that Ser361 phosphorylation participates in TXNIP‐mediated cell cycle arrest. In addition, the C‐arrestin domain may also play an important role in cell cycle arrest. We also showed that phosphorylation at Ser361 may be important for the association of TXNIP with JAB1 and that the C‐arrestin domain is necessary for the nuclear localization of this molecule. Collectively, these studies reveal that TXNIP participates in cell cycle regulation through association with regulatory proteins, especially JAB1, and that C‐arrestin‐dependent nuclear localization is important for this function. This work may facilitate the development of a new cancer therapy strategy that targets TXNIP as a key molecule inhibiting cancer cell growth via cell cycle blockade at the G1/S checkpoint.

**Abbreviations**
AcGFP: *Aequorea coerulescens* green fluorescent protein
CaMK: calmodulin‐dependent kinase
Cdk: cyclin‐dependent kinase
GST: glutathione S‐transferase
HRP: horse radish peroxidase
JAB1: Jun activation domain‐binding protein 1
LC‐MS/MS: liquid chromatography–mass spectrometry
MAPK: mitogen‐activated protein kinase
PKA: protein kinase A
PKC: protein kinase C
TXNIP: thioredoxin interacting protein

Thioredoxin interacting protein (TXNIP), also called thioredoxin‐binding protein‐2 or vitamin D_3_ up‐regulated protein 1, was originally identified as a molecule up‐regulated in HL‐60 leukemia cells by 1,25‐dihydroxyvitamin D_3_ treatment 1. It has been recently recognized as a tumor suppressor protein based on a number of clinical and experimental reports. For example, pathological analyses have revealed that its expression is reduced in various tumor tissues, including breast, lung, stomach, and colon tumors 2, 3. In addition, *in vitro* studies indicate that TXNIP overexpression can inhibit the proliferation of stomach cancer and leukemia cells 4, 5. Furthermore, TXNIP expression is related to the prognosis of lymphoma and breast cancer 6, 7 and melanoma metastasis 8. More interestingly, both mice with spontaneous mutation and mice with knockout of the *TXNIP* gene showed dramatically increased incidence of hepatocellular carcinoma 9, 10. Although these observations raised the possibility of TXNIP as a target for cancer therapies, a clinical application focusing on this molecule has not been developed so far. Molecular analysis of the TXNIP tumor‐suppressive effect could lead to an understanding of the mechanisms of tumor progression or to development of novel cancer therapies.

TXNIP has two independent mechanisms for its tumor‐suppressive effect, depending on the cell type and the environment. Firstly, its function depends on apoptosis induction through the inhibition of thioredoxin activity in some cell types 2, 11, 12, 13. Secondly, TXNIP induces cell cycle arrest at the G1/S checkpoint through the thioredoxin‐independent pathway in several tumor cell lines 14, 15, 16, 17, 18. The cell cycle is strictly regulated by the expression and phosphorylation of cyclins and cyclin‐dependent kinases (Cdks), and transition from G1 to S phase is accelerated by the cyclin E–Cdk2 complex. The activity of this complex is regulated by p27^kip1^, one of the Cdk inhibitory molecules 19. Due to its inhibitory function in cell cycle progression, p27^kip1^ is induced or activated by various growth arrest signals 20. The function of p27^kip1^ is inhibited by association with a shuttle protein, Jun activation domain‐binding protein 1 (JAB1), in the nucleus, since the p27^kip1^–JAB1 complex translocates to the cytoplasm for subsequent ubiquitin‐dependent degradation of p27^kip1^ 21, 22. TXNIP associates with JAB1 and this leads to the dissociation of p27^kip1^ and JAB1. Therefore, when a sufficient amount of TXNIP is present in the nucleus, nuclear export of p27^kip1^ is inhibited, and p27^kip1^ stably localizes in the nucleus and effectively inhibits the transition from G1 to S phase 23. These reports support the idea that TXNIP is a key molecule during the regulation of the cell cycle via association with JAB1, and further molecular analysis is necessary to understand the tumor‐suppressive effect of TXNIP in detail.

It has been reported that Thr349 and Ser361 of TXNIP are phosphorylated in HeLa cells during the G1 stage of the cell cycle 24; however, the physiological significance of phosphorylation at these sites has not been reported. Another structural feature of TXNIP is α‐arrestin, which contains two arrestin domains (N‐arrestin and C‐arrestin). Although prototype arrestins (visual arrestin and β‐arrestin) are key regulators of receptor signaling, the functions of the arrestin domains in α‐arrestin remain unclear 25.

Here, we elucidate molecular events concerning cell cycle regulation by TXNIP. We show phosphorylation of TXNIP by p38 mitogen‐activated protein kinase (MAPK), a signaling molecule that has various functions in cellular responses including cell cycle regulation 26, 27. Moreover, we analyzed the role of TXNIP phosphorylation at Ser361 and the C‐arrestin domain during cell cycle blockade at the G1/S transition. These studies could provide a new strategy for cancer therapy that targets TXNIP as a key molecule, inhibiting cancer cell growth via cell cycle blockade at the G1/S checkpoint.

## Materials and methods

### Plasmid constructs

Expression vectors pFLAG‐TXNIP, pmyc‐p27^kip1^, pJAB1‐V5, and pAcGFP‐TXNIP were described previously 15. Human Cdk2, Cdk4, cyclin B1, cyclin D1, cyclin E splicing variant (GenBank NM_001238), p38 MAPK, protein kinase A (PKA), and protein kinase C (PKC) cDNA were cloned into the pEF/V5‐His mammalian expression vector (Thermo Fisher Scientific (Invitrogen), Waltham, MA, USA). Human p27^kip1^, JAB1, Cdk2, and cyclin E cDNA were cloned into pGEX‐6P‐1 (GE Healthcare, Little Chalfont, UK) and used as *Escherichia coli* expression vectors for glutathione *S*‐transferase (GST) fusion proteins. The human TXNIP cDNA was cloned into pCold DNA (Takara Bio, Kusatsu, Shiga, Japan) and used as the pColdHis‐TXNIP *E. coli* expression vector. Point or deletion mutants of pFLAG–TXNIP or *Aequorea coerulescens* green fluorescent protein (pAcGFP)–TXNIP were prepared using PrimeSTAR Mutagenesis Basal Kit (Takara Bio). pFLAG–TXNIP (T349A) and pAcGFP–TXNIP (T349A) have a single point mutation from threonine to alanine at amino acid 349. pFLAG–TXNIP (S361A) and pAcGFP–TXNIP (S361A) have a single point mutation from serine to alanine at amino acid 361. pFLAG–TXNIP (delN) and pAcGFP–TXNIP (delN) have an amino acid 10–152 deletion, and pFLAG–TXNIP (delC) and pAcGFP–TXNIP (delC) have an amino acid 174–296 deletion.

### Protein purification and *in vitro* binding assay

The *E. coli* BL21 strain was transformed with each expression plasmid and was cultured at 37 °C until *D*_600_ reached 0.5. To express GST‐fusion proteins, isopropyl β‐d‐1‐thiogalactopyranoside was supplied at 0.5 mm and then incubated at 25 °C for 4 h. To express His–TXNIP, the culture was set at 4 °C for 30 min, isopropyl β‐d‐1‐thiogalactopyranoside was supplied at 0.5 mm, and then the culture was further incubated at 4 °C for 4 h. Each GST‐fusion protein was purified using Glutathione Sepharose 4B (GE Healthcare). His‐tagged TXNIP protein was purified using Ni‐NTA agarose (Qiagen, Venlo, Netherlands). Purified proteins were separated by SDS/PAGE and stained with Coomassie Brilliant Blue R‐250. The *in vitro* binding analysis of TXNIP and each protein was performed as follows. Two micrograms of each GST‐fusion protein and 200 ng His–TXNIP were incubated in a 500 μL binding buffer (20 mm Tris/HCl pH 7.5, 150 mm NaCl, 0.1% Triton X‐100, 20 μL Glutathione Sepharose 4B) at 4 °C for 3 h and was washed 3 times with 1 mL wash buffer (20 mm Tris/HCl pH 7.5, 150 mm NaCl, 0.1% Triton X‐100). The affinity of TXNIP and GST‐fusion protein was analyzed by western blot for TXNIP.

### Cell culture and transfection

COS‐7 cells and HuH‐7 cells were maintained in Dulbecco's modified Eagle's medium (Sigma‐Aldrich, St Louis, MO, USA) supplemented with 10% fetal calf serum. Transient transfection was performed using Fugene 6 transfection reagent (Roche Diagnostics, Mannheim, Germany) for COS‐7 cells, and using X‐tremeGENE HP DNA Transfection Reagent (Sigma‐Aldrich) following the manufacturer's protocol.

### Immunoprecipitation

Immunoprecipitations were performed as described previously 15. Materials used are as follows: anti‐FLAG M2 affinity gel (Sigma‐Aldrich), anti‐myc agarose affinity gel (Sigma‐Aldrich), anti‐V5 agarose affinity gel (Sigma‐Aldrich), Protein G Sepharose 4 fast flow (GE Healthcare), and anti‐TXNIP antibody (MBL, Nagoya, Aichi, Japan). For immunoprecipitation with antibody‐conjugated affinity gel, 1 mg whole‐cell protein was incubated with 20 μL affinity gel at 4 °C for 2 h. For immunoprecipitation with anti‐TXNIP antibody, 2 mg whole‐cell protein was incubated with the antibody at 4 °C for 1 h, then 30 μL Protein G Sepharose was added, and it was additionally incubated for 2 h.

### Western blot analysis

Western blot analyses were performed as described previously 15. Primary antibodies used for western blot and dilution are as follows: anti‐FLAG M2–horse radish peroxidase (HRP) antibody (Sigma‐Aldrich, 1 : 1000), anti‐myc–HRP antibody (Thermo Fisher Scientific, 1 : 5000) and anti‐V5–HRP antibody (Thermo Fisher Scientific, 1 : 5000), anti‐Lamin A/C antibody (Cell Signaling Technology, Danvers, MA, USA, 1 : 1000), anti‐glyceraldehyde‐3‐phosphate dehydrogenase (Cell Signaling Technology, 1 : 500), anti‐p27^kip1^ antibody (MBL, 1 : 1000), anti‐JAB1 antibody (Santa Cruz Biotechnology, Dallas, TX, USA, 1 : 200), anti‐Cdk2 antibody (Santa Cruz Biotechnology, 1 : 200), and anti‐cyclin E antibody (MBL, 1 : 1000). Anti‐mouse IgG HRP‐linked and anti‐rabbit IgG HRP‐linked (Cell Signaling Technology, 1 : 5000) secondary antibodies were used. Data were analyzed using lumivision analyzer 400 software (Aisin Seiki, Kariya, Aichi, Japan).

### Phosphoprotein analysis

HuH‐7 cells were treated with 50 mm d‐allose (Rare Sugar Research Center, Kagawa University, Kagawa, Japan) for 48 h, and cell lysate was immunoprecipitated against anti‐TXNIP. COS‐7 cells were transfected with FLAG–TXNIP expression plasmid, and cell lysate was immunoprecipitated with anti‐FLAG agarose as described above. The immunoprecipitates were separated by SDS/PAGE. To visualize phosphoprotein, the gel was stained using the Pro‐Q Diamond phosphoprotein gel stain (Thermo Fisher Scientific (Invitrogen)) following the manufacturer's protocol. The p38 MAPK activity was inhibited by pre‐incubation in 500 nm LY2228820 (Selleck Chemicals, Houston, TX, USA) for 2 h before preparing the cell lysate. The samples for liquid chromatography–mass spectrometry (LC‐MS/MS) analyses were prepared by in‐gel‐digestion of the proteins by trypsin, chymotrypsin, and aspartic protease. The phosphorylation state was analyzed by LC‐MS/MS using Micromass Q‐TOF (Waters, Milford, MA, USA), followed by a Mascot search (Matrix Science, Boston, MA, USA).

### *In vitro* kinase assay

One milligram of purified FLAG–TXNIP was incubated at 30 °C for 10 min in kinase reaction mix (2.96 MBq·mL^−1^ γ‐[^32^P]ATP, 2 μg·mL^−1^ 200 μm ATP, and 0.2 mm dithiothreitol). For the p38 MAPK reaction, 25 mm Tris/HCl pH 7.5, 10 mm magnesium acetate, 20 μm EGTA, and 10 ng·μL^−1^ p38 MAPK (Cell Signaling Technology) were added to the kinase reaction mix. For the calmodulin‐dependent kinase (CaMK) reactions, 50 mm HEPES pH 7.5, 10 mm magnesium acetate, 1 mm CaCl_2_, 4 μm calmodulin, and 2 ng·μL^−1^ CaMKI or CaMKIV (gift from H. Tokumitsu, Okayama University) were added to the kinase reaction mix. For the PKA reaction, 50 mm Tris/HCl pH 7.5, 10 mm MgCl_2_, 2 μm cyclic adenosine monophosphate, and 6.7 ng·μL^−1^ PKA (Promega, Madison, WI, USA) were added to the kinase reaction mix. For the PKC reaction, 20 mm Tris/HCl pH 7.5, 5 mm MgCl_2_, 1 mm CaCl_2_, 320 ng·μL^−1^ phosphatidylserine, 32 ng·μL^−1^ diacylglycerol, and 3 ng·μL^−1^ PKC (Promega) were added to the kinase reaction mix. The reaction products were separated by SDS/PAGE. The gel was dried and radioactive phosphorylated proteins were detected by autoradiography.

### Cell cycle analysis

HuH‐7 cells were transfected with expression plasmids for AcGFP‐fusion proteins and incubated for 48 h. Cells were fixed in 5.5% formaldehyde and treated with 200 μg·mL^−1^ RNase A (Sigma‐Aldrich) and 100 μg·mL^−1^ propidium iodide (Wako Pure Chemical Industries, Osaka, Japan) using Intraprep Permeabilization Reagent (Beckman Coulter, Brea, CA, USA) following the manufacturer's protocol. Flow cytometry analysis was performed for GFP/propidium iodide‐positive cells using a Cytomics FC 500 (Beckman Coulter).

### Immunofluorescence analysis

Cell culture and immunostaining were performed as described previously 17. Anti‐FLAG M2 (Sigma‐Aldrich, 1 : 500) was used as the primary antibody, and Alexa Fluor 488 anti‐mouse IgG (Thermo Fisher Scientific (Invitrogen), 1 : 400) was used as the secondary antibody. Nuclear staining was performed by incubation in 1 μg·mL^−1^ 4′,6‐diamidino‐2‐phenylindole (Dojindo Laboratories, Kumamoto, Japan). Signals were analyzed with a confocal laser scanning microscope, Radiance 2100 (Bio‐Rad, Hercules, CA, USA).

## Results

### TXNIP association with cell cycle regulatory proteins

**Figure 1:**
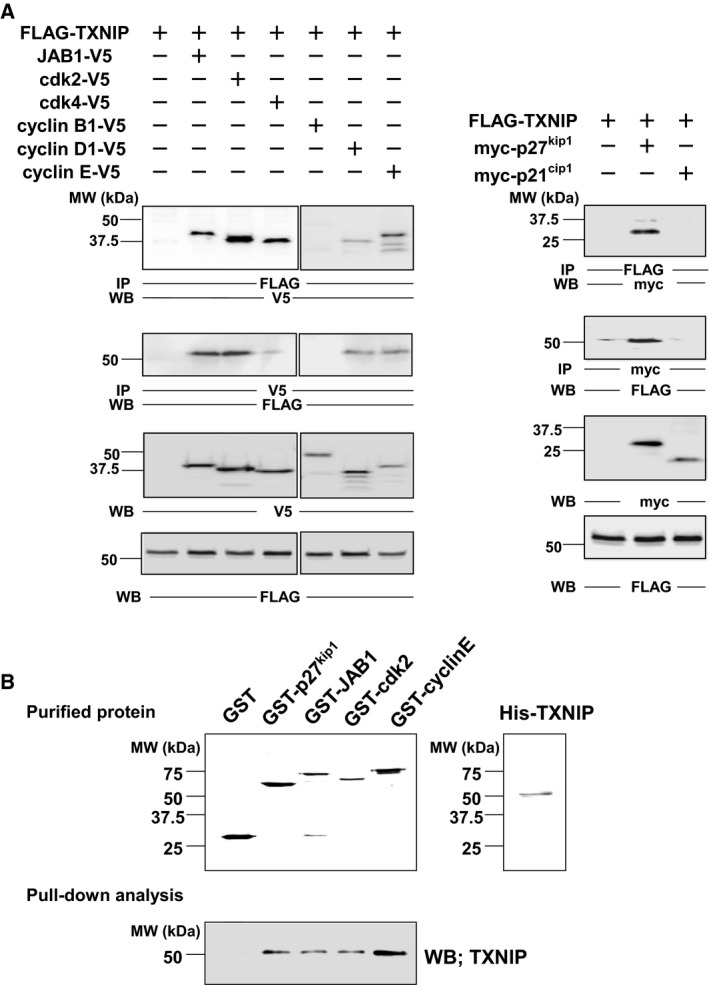
Interaction of TXNIP and cell cycle regulatory proteins *in vitro* and *in vivo*. (A) *In vivo* interaction analysis by immunoprecipitation. COS‐7 cells were transfected with plasmids as indicated. Immunoprecipitation followed by western blot analysis showed the interaction of TXNIP to each of p27^kip1^, JAB1, Cdk2, and cyclin E. (B) *In vitro* interaction analysis. GST‐p27^kip1^, GST–JAB1, GST–Cdk2, GST–cyclin E, and His–TXNIP were expressed in *E. coli* and affinity‐purified. Purified proteins were separated by SDS/PAGE, and the gels were stained with Coomassie Brilliant Blue R‐250. Interaction of purified His–TXNIP with each protein was analyzed, followed by western blot for TXNIP. IP, immunoprecipitation; WB, western blot.

Our previous study showed that TXNIP inhibited cell cycle progression at the G1/S checkpoint, through interaction with p27^kip1^ and JAB1 15. To further understand the role of TXNIP during the cell cycle at the G1/S checkpoint, we analyzed *in vivo* association between TXNIP and cell cycle regulatory proteins by overexpressing these proteins in COS‐7 cells, followed by immunoprecipitation analysis. The results clearly showed the association of TXNIP with p27^kip1^ and JAB1 as previously reported 15, and additionally, with Cdk2 and cyclin E. TXNIP also associated with Cdk4 and cyclin D1 with relatively lower specificity. The association of TXNIP with neither cyclin B1 nor p21^cip1^ could be detected (Fig. 1A). It was not clear whether TXNIP associates with each of these proteins directly or indirectly through the association with other proteins, consisting of a protein complex. To clarify this issue, further *in vitro* binding analyses were performed using purified proteins. As a result, direct associations of TXNIP with p27^kip1^, JAB1, Cdk2, and cyclin E were observed (Fig. 1B).

### Phosphorylation of TXNIP by p38 MAPK

**Figure 2:**
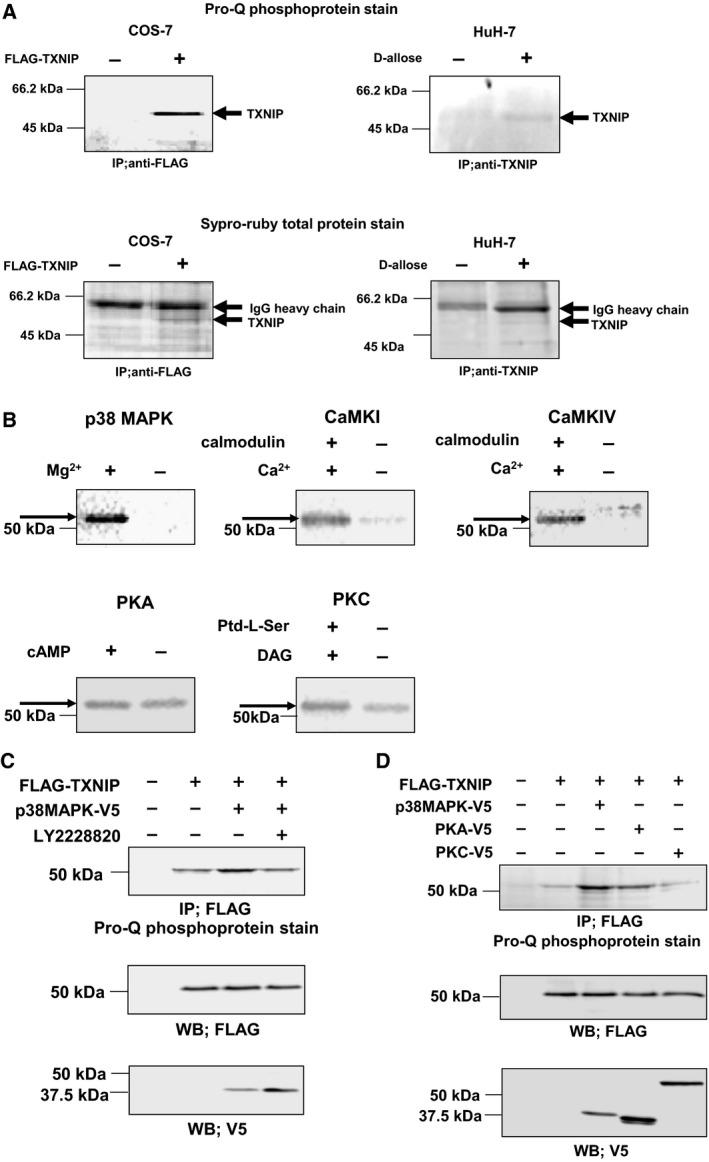
TXNIP is phosphorylated by p38 MAPK. (A) Phosphorylation of TXNIP in COS‐7 cells and HuH‐7 cells. COS‐7 cells were transfected with expression plasmid for FLAG–TXNIP, and cell lysate was immunoprecipitated with anti‐FLAG agarose gel. HuH‐7 cells were treated with 50 mm d‐allose for 48 h, and cell lysate was immunoprecipitated with anti‐TXNIP/protein G, and separated by SDS/PAGE. The phosphorylation of TXNIP was detected by Pro‐Q phosphoprotein gel stain. (B) *In vitro* phosphorylation analysis of TXNIP by autoradiography. Affinity‐purified FLAG–TXNIP protein was incubated in the presence of each kinase and γ‐[^32^P]ATP, and phosphorylated TXNIP was detected. (C) Phosphorylation of TXNIP by p38 MAPK in COS‐7 cells. COS‐7 cells were transfected with expression plasmid for FLAG–TXNIP and p38 MAPK–V5. Cells were pretreated with LY2228820 (500 nm; 2 h) as indicated. The phosphorylation of TXNIP was analyzed by immunoprecipitation followed by the Pro‐Q phosphoprotein gel stain. (D) Phosphorylation of TXNIP by kinases in COS‐7 cells. COS‐7 cells were cotransfected with expression plasmids for FLAG–TXNIP and each V5‐tagged kinase. The phosphorylation of TXNIP was analyzed by immunoprecipitation followed by Pro‐Q phosphoprotein gel stain.

As a next step, we analyzed TXNIP phosphorylation to elucidate its role in the cell cycle regulation. Since the NetPhosK Server analysis (CBS Prediction Servers, Technical University of Denmark) suggested that TXNIP contained multiple phosphorylation sites, we analyzed the phosphorylation state of FLAG–TXNIP overexpressed in COS‐7 cells with the result that TXNIP was clearly detected as a phosphoprotein. The phosphorylation of endogenous TXNIP was also detected using HuH‐7 treated with d‐allose, which up‐regulates TXNIP expression in this cell line (Fig. 2A). We then examined kinases that could phosphorylate TXNIP *in vitro*. Since the NetPhosK Server analysis predicted phosphorylation of TXNIP by CaMKs, PKA, PKC, and p38 MAPK, *in vitro* kinase assays of TXNIP by these enzymes were performed. As a result, we found that TXNIP was clearly and specifically phosphorylated by p38 MAPK, CaMKI, and CaMKIV, but PKA and PKC did not show any specific phosphorylation signal (Fig. 2B). Next, we examined the possibility of *in vivo* phosphorylation of TXNIP by p38 MAPK, by coexpressing these proteins in COS‐7 cells and subsequent phosphoprotein staining. The result showed that TXNIP was phosphorylated, and its phosphorylation level increased upon coexpression with p38 MAPK. The p38 MAPK inhibitor LY2228820 suppressed the enhancement of phosphorylation (Fig. 2C). The coexpression of TXNIP and PKA or PKC did not enhance the phosphorylation of TXNIP (Fig. 2D). Overall, these analyses suggested direct and specific phosphorylation of TXNIP by p38 MAPK.

### Phosphorylation of TXNIP at Ser361

**Figure 3:**
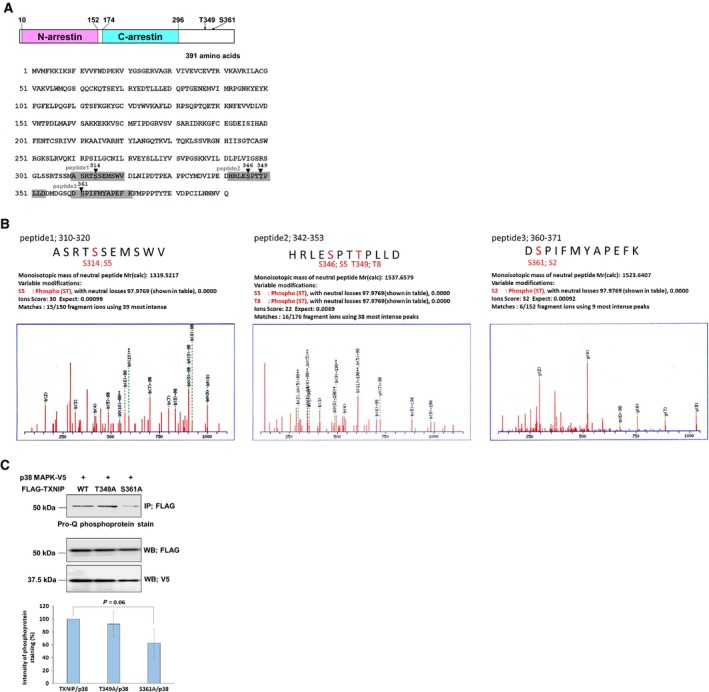
Analysis of TXNIP phosphorylation sites. (A) Structure of TXNIP. Two α‐arrestin domains and putative phosphorylation sites analyzed in this study are indicated. Phosphopeptides analyzed by LC‐MS/MS are indicated with gray boxes. (B) LC‐MS/MS analysis of TXNIP overexpressed in COS‐7 cells. Affinity‐purified FLAG–TXNIP protein was in‐gel digested by trypsin, chymotrypsin, and aspartic protease, and its phosphorylation state was analyzed by mass spectrometry. Phosphorylation of Ser314, Ser346, Thr349, and Ser361 was detected. (C) COS‐7 cells were transfected with each expression plasmid as indicated, and cell lysate was immunoprecipitated with anti‐FLAG agarose gel. The phosphorylation state of TXNIP and its mutants were analyzed using Pro‐Q phosphoprotein gel stain. The intensity of phosphorylation bands was shown as mean ± SD (*t* test, *n* = 4).

To identify phosphorylation sites in TXNIP, FLAG–TXNIP expressed in COS‐7 cells was analyzed by LC‐MS/MS. The results suggested that Ser314, Ser346, Thr349, and Ser361 were phosphorylated (Fig. 3A,B). An independent report showing quantitative phosphorylation analysis in HeLa cells implied that the phosphorylation of Thr349 and Ser361 occurred in G1‐ and M‐phase cells 24. Our result and that report suggest that Thr349 and Ser361 could be major phosphorylation sites, possibly taking part in cell cycle regulation. To elucidate this hypothesis, the phosphorylation state of TXNIP at these sites was analyzed by overexpressing FLAG–TXNIP wild‐type, FLAG–TXNIP (T349A), and FLAG–TXNIP (S361A) in COS‐7 cells. p38 MAPK was also overexpressed to enhance the phosphorylation level. As a result, S361A showed a lower phosphorylation level than wild‐type (62.6 ± 22.0%, *P* = 0.06, *n* = 4), while T349A showed a similar phosphorylation level to wild‐type (92.4 ± 19.6%, *n* = 4; Fig. 3C). This result suggests that Ser361 is one of the major phosphorylation sites of TXNIP upon coexpression with p38 MAPK in COS‐7 cells, and that no or little Thr349 is phosphorylated under the same conditions.

### Cell cycle analysis of TXNIP mutants

**Figure 4:**
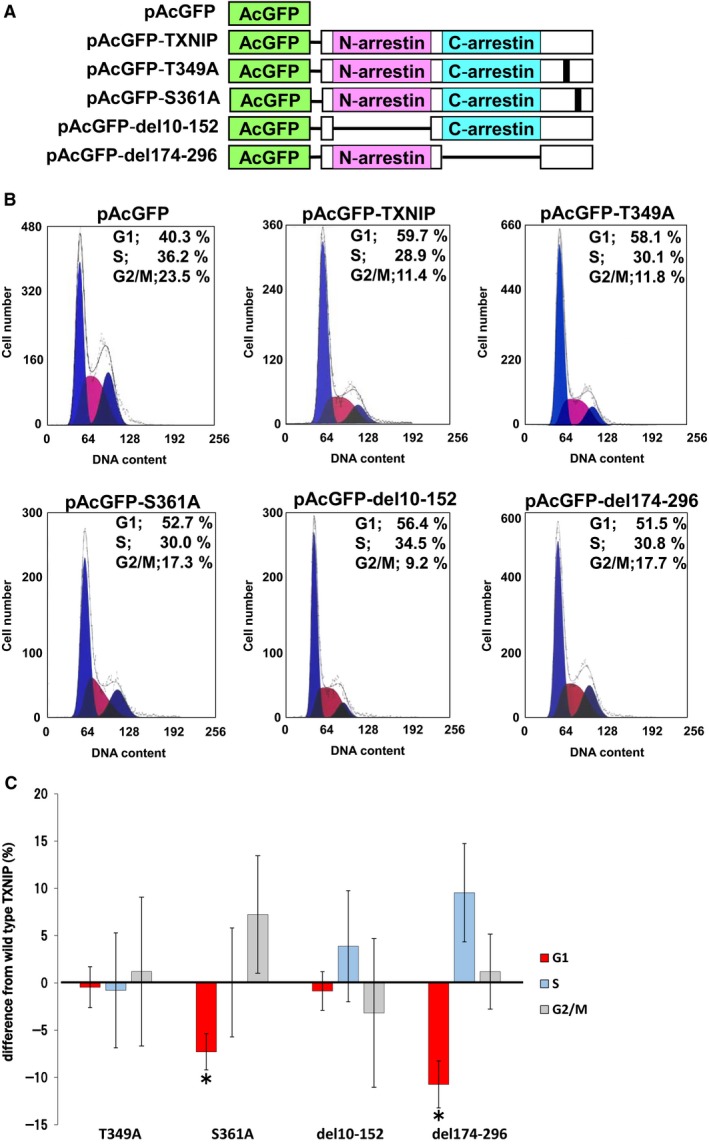
TXNIP Ser361 and C‐arrestin domain modulate cell cycle progression. (A) Expression plasmids for AcGFP (control) and its fusion protein with TXNIP (wild‐type or mutants) used for the cell cycle analysis. (B) HuH‐7 cells were transfected with each expression plasmid. After 48 h incubation, the percentage of cells in G1, G2/M, and S stage was calculated for AcGFP‐positive cells. (C) Statistical analysis of cell cycle. For each value, difference from the control (AcGFP) was calculated and shown as mean ± SD (*P <* *0.05 *vs* wild‐type TXNIP, *t* test, *n* = 4).

We next analyzed the cell cycle of hepatocellular carcinoma cell line HuH‐7 overexpressing pAcGFP–TXNIP and its mutants as illustrated in Fig. 4A. Since HuH‐7 cells do not express TXNIP at a level detectable by western blot 15, the effects of endogenous TXNIP were negligible in this experiment. The result of one experiment in each group is shown in Fig. 4B. When *Aequorea coerulescens* green fluorescent protein (AcGFP)–TXNIP is overexpressed, the number of cells in the G1 stage increased by more than 15% compared to the control cells (expressing AcGFP), which was consistent with our previous analysis 15. The results of four independent experiments were statistically analyzed and compared to the TXNIP wild‐type value as shown in Fig. 4C. Cells in G1 stage significantly decreased by 7.29 ± 1.92% compared to the wild‐type value among cells expressing the S361A mutant. Cells expressing del174–296, the deletion mutant of the C‐arrestin domain, also showed a significant reduction of cells in the G1 stage (10.7 ± 2.49%). Cells expressing T349A or del10–152 did not show a significant difference from cells expressing TXNIP wild‐type (−0.456 ± 2.16% and −0.858 ± 2.04%, respectively). These results suggest that both S361 phosphorylation and the C‐arrestin domain play important roles in the cell cycle arrest caused by TXNIP.

### S361A mutant showed lower association with JAB1

**Figure 5:**
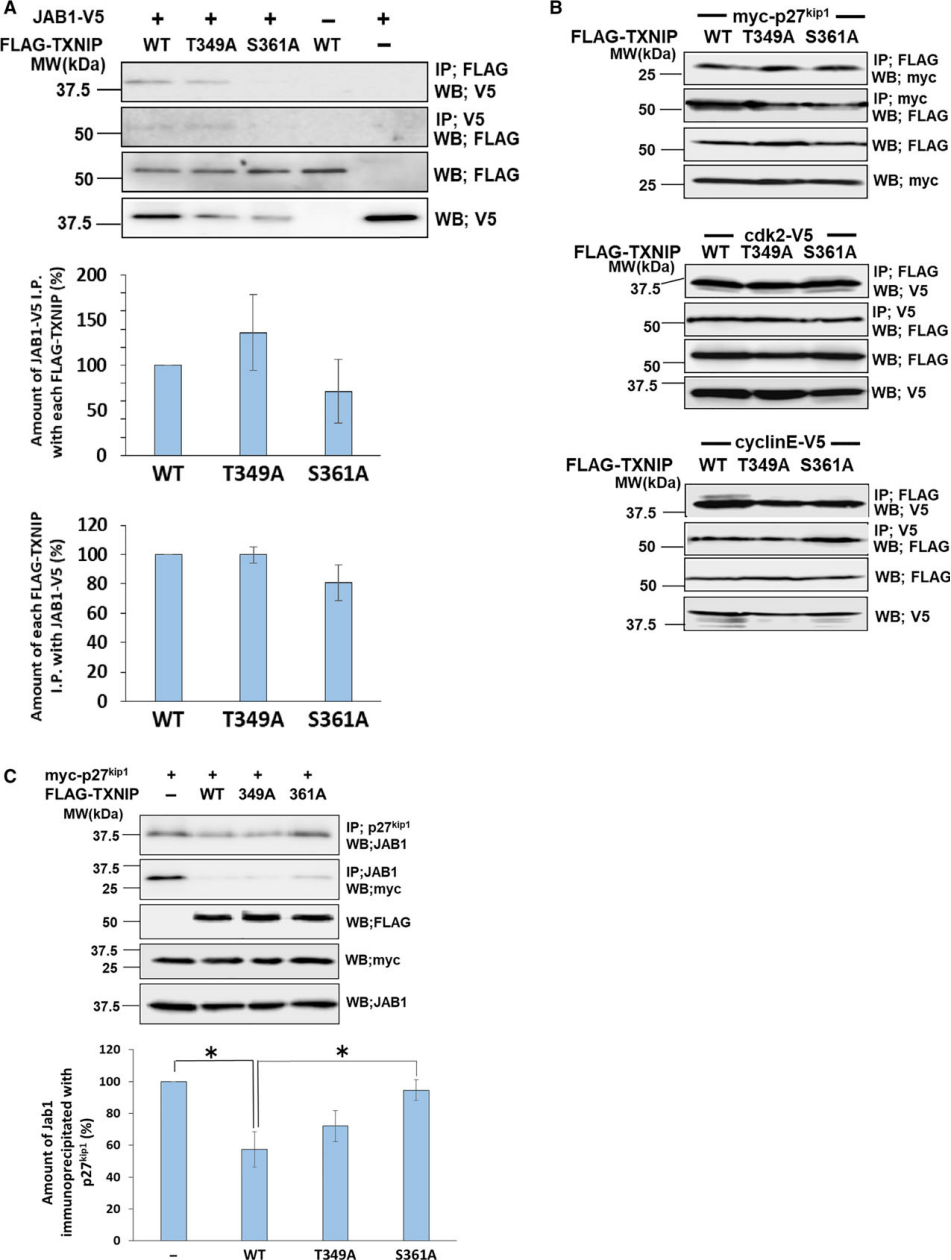
TXNIP phosphorylation at Ser361 promotes the interaction with JAB1. (A, B) COS‐7 cells were cotransfected by expression plasmids for FLAG–TXNIP (wild‐type (WT), FLAG–T349A or FLAG–S361A) and JAB1–V5 (A), myc‐p27^kip1^, Cdk2–V5, or cyclin E–V5 (B), as indicated. Protein complex was immunoprecipitated by anti‐V5, anti‐myc or anti‐FLAG agarose, and the protein interaction was analyzed by western blot analysis. Error bars in the graphs indicate mean ± SD. (C) COS‐7 cells were cotransfected by expression plasmids for myc‐p27^kip1^ and FLAG–TXNIP (wild‐type, FLAG–T349A or FLAG–S361A). Protein complex was immunoprecipitated by different types of antibodies as indicated in the figure. Western blot analysis was used to analyze the interaction of p27^kip1^ and JAB1. Signal densities of JAB1 immunoprecipitated with p27^kip1^ were analyzed. For each value, difference from the control (without TXNIP transfection) was calculated and expressed as mean ± SD (*P <* *0.05, *t* test, *n* = 3).

Next, we performed immunoprecipitation studies to analyze TXNIP association with cell cycle regulatory proteins. As a result, the S361A associated with JAB1 in less extent compared to the TXNIP wild‐type, while the T349A did not show much difference compared to the wild‐type (Fig. 5A). This result suggests that the Ser361 phosphorylation possibly takes part in the association of TXNIP with JAB1. Wild‐type TXNIP, T349A, and S361A associated with each of p27^kip1^, cyclin E, or Cdk2 at similar levels (Fig. 5B). We further examined the association between JAB1 and p27^kip1^ upon overexpression of TXNIP and its mutants. The immunoprecipitation study showed that the overexpression of wild‐type TXNIP reduced the amount of JAB1 immunoprecipitated with p27^kip1^ (57.5 ± 11.2% compared to the control). Overexpression of T349A showed some reduction of the association between JAB1 and p27^kip1^ (72.0 ± 9.7% compared to the control), and that of S361A showed a slight reduction (94.6 ± 6.6% compared to the control; Fig. 5C).

### C‐Arrestin domain is necessary for nuclear localization

**Figure 6:**
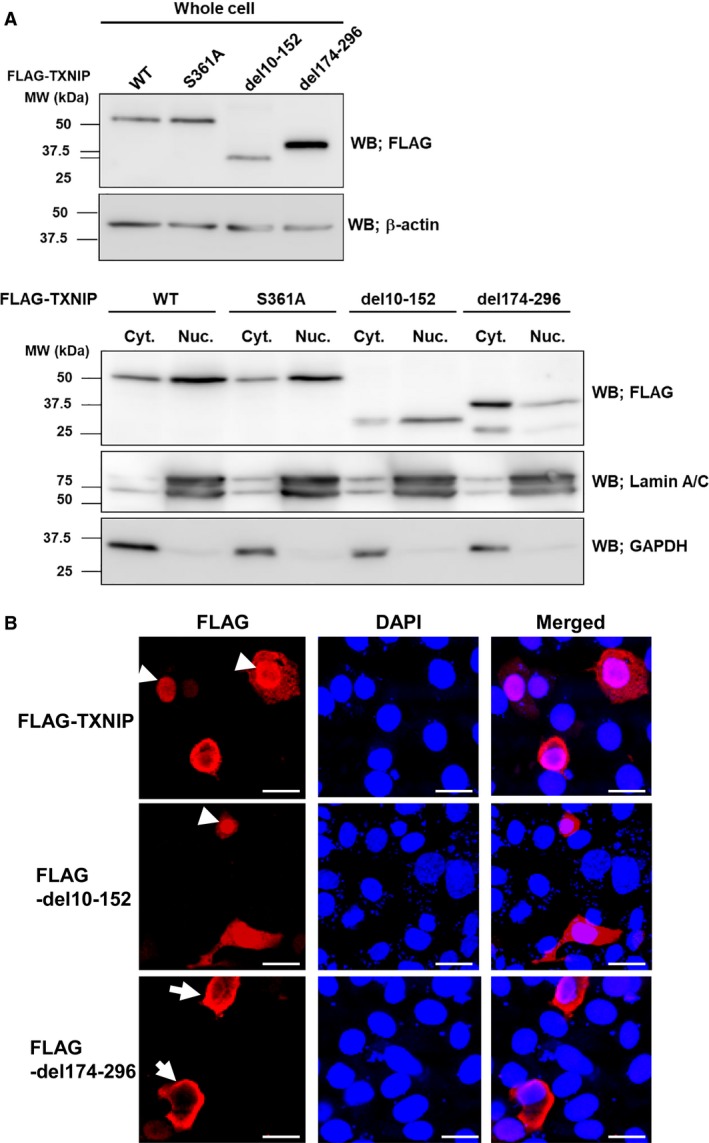
TXNIP C‐arrestin domain is necessary for nuclear localization. (A) HuH‐7 cells were transfected with expression plasmids for TXNIP wild‐type (WT) or its mutants. Whole‐cell lysates, cytoplasmic and nuclear fractions of the transfected HuH‐7 cells were prepared. Western blot analysis showed that wild‐type and S361A mutant mainly localized in nuclei, while del174–296 mutant dominantly localized in the cytoplasm. Del10–152 mutant localized in both nuclei and cytoplasm. (B) Immunofluorescence analysis showed similar results to that of western blot analysis. The localizations of protein in the nuclei and in the cytoplasm are indicated with arrowheads and arrows, respectively. Scale bars: 20 μm. DAPI, 4′,6‐diamidino‐2‐phenylindole; GAPDH, glyceraldehyde 3‐phosphate dehydrogenase.

Next we analyzed how the C‐arrestin domain in TXNIP participates in cell cycle regulation. We prepared whole‐cell lysate, the cytoplasmic fraction, and the nuclear fraction from cells expressing wild‐type TXNIP or its mutants, followed by the western blot analysis. The blot for whole‐cell lysates detected each protein at the expected size. The result for fractionated proteins showed that 57.7 ± 4.9% of wild‐type TXNIP localized in the nuclei, and 42.3 ± 4.9% localized in the cytoplasm. The S361A mutant localized in the nuclei at 68.3 ± 5.5%, and in the cytoplasm at 31.7 ± 5.5%. The del10–152 mutant localized in the nuclei at 52.5 ± 1.68%, and in the cytoplasm at 47.5 ± 1.68%. Surprisingly, the del174–296 mutant dominantly localized in cytoplasm (80.2 ± 8.6%), and only 19.8 ± 8.6% localized in the nuclei (Fig. 6A). Immunofluorescence studies also showed that wild‐type TXNIP, S361A, and del10–152 localized both in the nucleus and in the cytoplasm, while del174–296 dominantly localized in the cytoplasm (Fig. 6B). These results suggest that the C‐arrestin domain is necessary for nuclear localization of TXNIP. Taken together with the results of cell cycle analysis, nuclear localization of TXNIP is a prerequisite for the function of this protein in cell cycle regulation.

### Role of TXNIP in p27^kip1^ stability

**Figure 7:**
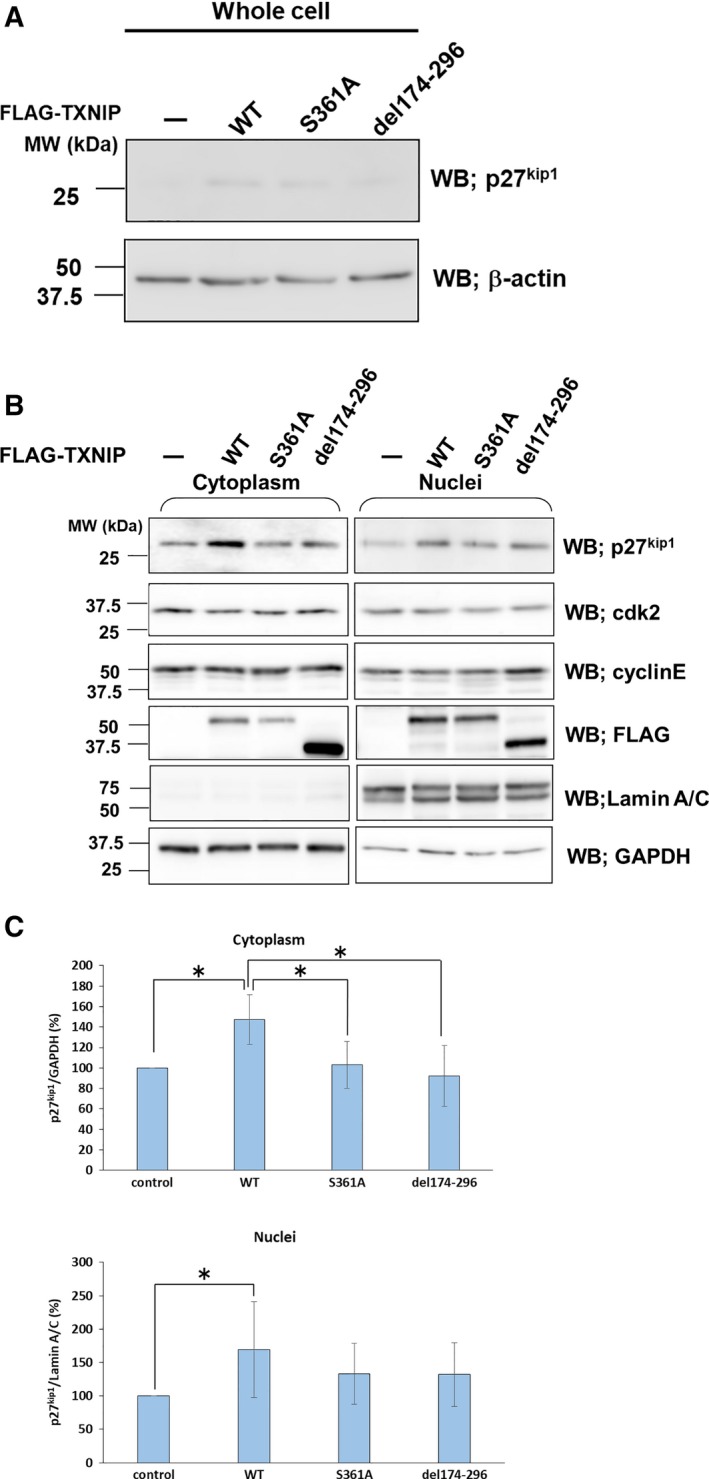
Phosphorylation at Ser361 and C‐arrestin domain regulates the amount of p27^kip1^. HuH‐ 7 cells were transfected with TXNIP or its mutants in the expression plasmids. Whole‐cell lysates (A), cytoplasmic and nuclear fractions (B) of the transfected HuH‐7 cells were prepared for western blots. The antibodies used in Western blots are indicated in the figure. (C) Statistical analysis of western blots for p27^kip1^. Amount of p27^kip1^ in the transfected cells (TXNIP wild‐type or its mutants) was compared with that of non‐transfected cells for cytoplasm and nuclei. The relative amounts of p27^kip1^ are expressed as mean ± SD (*P <* *0.05, *t* test, *n* = 4). GAPDH, glyceraldehyde 3‐phosphate dehydrogenase; WT, wild‐type.

The present data revealed that the phosphorylation at Ser361 and the C‐arrestin domain of TXNIP are important for cell cycle regulation via independent mechanisms. We further analyzed the role of TXNIP Ser361 and the C‐arrestin domain for the stabilization of p27^kip1^. Western blot for whole‐cell protein suggested an increase of p27^kip1^ upon TXNIP overexpression. Overexpression of S361A or del174–296 increased p27^kip1^ at a lower level (Fig. 7A). The western blots for fractionated protein showed that the amount of p27^kip1^ increased to 147.3 ± 24.5% in cytoplasm, and to 169.7 ± 71.7% in nuclei, in cells overexpressing TXNIP, compared to that in control cells. This result supports the previous report showing that TXNIP contributes to the stability of p27^kip1^ 23. Overexpression of S361A and del174–296 did not cause significant increase of p27^kip1^ compared to the no‐transfect control, either in cytoplasm or in nuclei. Overexpression of TXNIP or its mutants did not change the amount of Cdk2 and cyclin E (Fig. 7B,C). These data indicate that both phosphorylation at Ser361 and the C‐arrestin domain play a role in p27^kip1^ stabilization by TXNIP.

## Discussion

**Figure 8:**
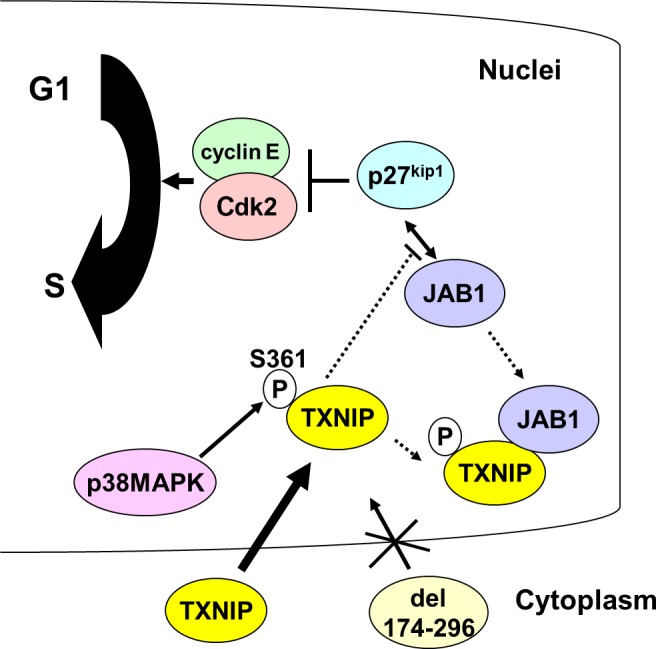
Possible role of Ser361 phosphorylation and C‐arrestin domain during cell cycle regulation. TXNIP is phosphorylated at Ser361 by p38 MAPK. This phosphorylation could facilitate binding of TXNIP and JAB1. As a result, the association between JAB1 and p27^kip1^ could weaken, and p27^kip1^ stably functions as a negative regulator of cyclin E–Cdk2. TXNIP C‐arrestin domain is responsible for its nuclear localization, which is a prerequisite for the function of TXNIP in cell cycle regulation.

Cell cycle progression is regulated by phosphorylation of many regulatory proteins. For example, it is well documented that Cdks and cyclins are phosphorylated at each specific stage. Although TXNIP phosphorylation at Thr349 and Ser361 had been previously reported 24, the physiological significance of its phosphorylation at these sites has not been clarified so far. The present data reveal that Ser361 is one of the major phosphorylation sites, and more importantly, this phosphorylation could relate to the cell cycle arrest caused by TXNIP. Our data also imply that the S361A mutant has lower association with JAB1 compared to the wild‐type TXNIP, and that TXNIP interrupts the association between JAB1 and p27^kip1^. Taken together with the results of cell cycle analysis, the Ser361 phosphorylation of TXNIP could take part in the cell cycle arrest at the G1/S transition through the association with JAB1, resulting in a reduction of the association between JAB and p27^kip1^. Therefore, the Ser361 phosphorylation of TXNIP possibly plays an important role in cell cycle arrest through the association with JAB1 (Fig. 8).

In addition to S361, other amino acids should be phosphorylated because the S361A mutant was still phosphorylated. The *in vitro* phosphorylation of TXNIP by CaMKI and CaMKIV also suggests the existence of unidentified phosphorylation sites. Our preliminary analyses indicate that both double mutant TNXIP (T349A/S361A) and the multiple mutant (S346A/T348A/T349A/S361A) show a similar phosphorylation level to the S361 mutant. These results support the idea that none of S346, T348, and T349 is highly phosphorylated. Further mutation and phosphorylation analysis would be necessary to identify other phosphorylation sites and their roles in cell cycle regulation.

Here we report the possible role of TXNIP phosphorylation at S361 with regard to its association with JAB1. The pull‐down assay using proteins expressed in *E. coli* suggested the association of unphosphorylated TXNIP with JAB1, showing a discrepancy with the results of immunoprecipitation analysis. It is possible that unphosphorylated TXNIP has relatively low association with JAB1 and it can associate with JAB1 only in concentrated conditions as observed in the pull‐down assay. The phosphorylation at S361 in TXNIP could enhance the association with JAB1 *in vivo*.

It has been reported that TXNIP is up‐regulated through the activation of p38 MAPK in vascular smooth muscle cells, endothelial cells, and mesangial cells 28, 29, 30, 31. Independently, we have preliminarily observed the activation of p38 MAPK upon the stimulation by d‐allose, a monosaccharide that dramatically up‐regulates TXNIP, in HuH‐7 cells (data not shown), and our present data show direct phosphorylation of TXNIP by p38 MAPK *in vitro* and possibly *in vivo*. In addition, the p38 MAPK pathway has been reported to regulate the cell cycle 26, 27. Taken together, TXNIP possibly regulates the cell cycle through up‐regulation and activation by p38 MAPK. The role of p38 MAPK in the association of TXNIP with JAB1 or in the regulation of p27^kip1^ activity is unclear so far. The present results suggest the phosphorylation of S361A by p38 MAPK, and lower association of S361A with JAB1 compared to the wild‐type TXNIP in COS‐7 cells, which express endogenous p38 MAPK. Collectively endogenous p38 MAPK possibly plays a regulatory role in the association of TXNIP with JAB1 and thereby regulates the p27^kip1^ activity.

The present results show the association of TXNIP with Cdk2 and cyclin E. Cdk4, cyclin B and cyclin D show relatively low association with TXNIP. The cyclin E–Cdk2 complex specifically accelerates cell cycle progression from G1 to S phase. The cyclin D–Cdk4 complex participates in the regulation of the G0/G1 transition of the cell cycle, and cyclin B plays a role in mitosis. Taken together, TXNIP could specifically associate with the cyclin E–Cdk2 complex and participate in the regulation of the G1/S transition. However, the role of TXNIP in the cyclin E–Cdk2 complex has not been analyzed. Since the overexpression of TXNIP does not change the protein level of cyclin E or Cdk2, TXNIP does not regulate the amount of these proteins. Rather TXNIP possibly regulates the activity of the cyclin E–Cdk2 complex.

The result in Fig. 5C showed that TXNIP interrupted the association of p27^kip1^ and JAB1. In addition, Fig. 7 indicated that the protein level of p27^kip1^ was increased by the overexpression of TXNIP wild‐type, and the amount of p27^kip1^ was close to the control level in S361A‐ or del174–296‐overexpressing cells. These data imply that Ser361 and the C‐arrestin domain in TXNIP contribute to p27^kip1^ stabilization. However, we could not observe a significant difference in the nuclear p27^kip1^ amount between TXNIP‐overexpressing cells and each of S361A‐ and del174–296‐overexpressing cells. This result could be due to the rapid turnover of both p27^kip1^ and TXNIP caused by ubiquitin‐dependent degradation. Further studies would be needed to confirm that both phosphorylation at Ser361 and the C‐arrestin domain play certain roles in the p27^kip1^ stabilization caused by TXNIP.

While the arrestin domains in visual arrestin and β‐arrestin participate in G‐protein‐mediated signal transduction pathways 32, the functions of the arrestin domains in α‐arrestin, including that of TXNIP, remain unclear 25. Here, we show that amino acids 174–296 in TXNIP, corresponding to the C‐arrestin domain, are necessary for both nuclear localization and cell cycle regulatory function of TXNIP in HuH‐7 cells. Therefore, we hypothesize that the del174–296 mutant cannot participate in the cell cycle regulation because it cannot be translocated from cytoplasm to nuclei. Deletion of amino acids 10–152, corresponding to the N‐arrestin domain, did not have a significant effect on the cell cycle, and the western blot analysis and the immunofluorescence analysis clearly showed that del10–152 localized in both nuclei and cytoplasm. These findings agree on the point that TXNIP nuclear localization is a prerequisite for cell cycle regulation by this protein. Interestingly, Nishinaka *et al*. have previously reported that amino acids 1–227 of TXNIP were sufficient for the interaction with Rch1, a protein that is responsible for nuclear import of TXNIP. They also showed that this region is necessary for the nuclear localization of TXNIP in MCF‐7 cells 16. One explanation for this discrepancy is that amino acids 174–227, the overlapping region analyzed in this report and in the previous report, could be responsible for nuclear localization. Another possibility is that regulation of TXNIP localization depends on cell type. Further cell cycle analysis using a deletion mutant of this region, together with an interaction analysis with Rch1, would be necessary to clarify this issue.

Although TXNIP is known as a tumor suppressor, it has not been applied in any cancer therapy yet. Our previous work has shown that d‐allose, a monosaccharide rarely present in nature, is a strong inducer of TXNIP and can inhibit cell proliferation in various cancer cells 15, 17, 18. The present work elucidates the mechanism of TXNIP function in cell cycle regulation and may contribute to the establishment of a new strategy of cancer therapy. d‐Allose is one of the potential therapeutics, and we have already shown that this potential strategy is effective both in cultured cells 33 and in model mice 34, 35, 36, 37. Further translational research is necessary to strengthen this promising strategy.

## Author contributions

KK, FY, and MT conceived and designed the project, KK, YD, CN, YH, IT, and NH acquired the data, KK and FY analyzed and interpreted the data, and KK, AH, AK and MT wrote the paper.

## Conflict of interest

The authors declare no conflict of interest.

## References

[1] Chen KS and DeLuca HF (1994) Isolation and characterization of a novel cDNA from HL‐60 cells treated with 1,25‐dihydroxyvitamin D‐3. Biochem Biophys Acta 1219, 26–32. 8086474 10.1016/0167-4781(94)90242-9

[2] Butler LM , Zhou X , Xu WS , Scher HI , Rifkind RA , Marks PA and Richon VM (2002) The histone deacetylase inhibitor SAHA arrests cancer cell growth, up‐regulates thioredoxin‐binding protein‐2, and down‐regulates thioredoxin. Proc Natl Acad Sci USA 99, 11700–11705. 12189205 10.1073/pnas.182372299 PMC129332

[3] Ikarashi M , Takahashi Y , Ishii Y , Nagata T , Asai S and Ishikawa K (2002) Vitamin D3 up‐regulated protein 1 (VDUP1) expression in gastrointestinal cancer and its relation to stage of disease. Anticancer Res 22, 4045–4048. 12553030

[4] Han SH , Jeon JH , Ju HR , Jung U , Kim KY , Yoo HS , Lee YH , Song KS , Hwang HM , Na YS *et al* (2003) VDUP1 upregulated by TGF‐beta1 and 1,25‐dihydorxyvitamin D3 inhibits tumor cell growth by blocking cell‐cycle progression. Oncogene 22, 4035–4046. 12821938 10.1038/sj.onc.1206610

[5] Shin D , Jeon JH , Jeong M , Suh HW , Kim S , Kim HC , Moon OS , Kim YS , Chung JW , Yoon SR *et al* (2008) VDUP1 mediates nuclear export of HIF1alpha via CRM1‐dependent pathway. Biochem Biophys Acta 1783, 838–848. 18062927 10.1016/j.bbamcr.2007.10.012

[6] Tome ME , Johnson DB , Rimsza LM , Roberts RA , Grogan TM , Miller TP , Oberley LW and Briehl MM (2005) A redox signature score identifies diffuse large B‐cell lymphoma patients with a poor prognosis. Blood 106, 3594–3601. 16081686 10.1182/blood-2005-02-0487 PMC1895056

[7] Cadenas C , Franckenstein D , Schmidt M , Gehrmann M , Hermes M , Geppert B , Schormann W , Maccoux LJ , Schug M , Schumann A *et al* (2010) Role of thioredoxin reductase 1 and thioredoxin interacting protein in prognosis of breast cancer. Breast Cancer Res 12, R44. 20584310 10.1186/bcr2599 PMC2917039

[8] Goldberg SF , Miele ME , Hatta N , Takata M , Paquette‐Straub C , Freedman LP and Welch DR (2003) Melanoma metastasis suppression by chromosome 6: evidence for a pathway regulated by CRSP3 and TXNIP. Cancer Res 63, 432–440. 12543799

[9] Sheth SS , Bodnar JS , Ghazalpour A , Thipphavong CK , Tsutsumi S , Tward AD , Demant P , Kodama T , Aburatani H and Lusis AJ (2006) Hepatocellular carcinoma in Txnip‐deficient mice. Oncogene 25, 3528–3536. 16607285 10.1038/sj.onc.1209394

[10] Kwon HJ , Won YS , Suh YS , Jeon JH , Shao Y , Yoon SR , Chung JW , Kim TD , Kim HM , Nam KH *et al* (2010) Vitamin D3 upregulated protein 1 suppresses TNF‐α‐induced NF‐κB activation in hepatocarcinogenesis. J Immunol 185, 3980–3989. 20826751 10.4049/jimmunol.1000990

[11] Junn E , Han SH , Im JY , Yang Y , Cho EW , Um HD , Kim DK , Lee KW , Han PL , Rhee SG *et al* (2000) Vitamin D3 up‐regulated protein 1 mediates oxidative stress via suppressing the thioredoxin function. J Immunol 164, 6287–6295. 10843682 10.4049/jimmunol.164.12.6287

[12] Matsuoka S , Tsuchiya H , Sakabe T , Watanabe Y , Hoshikawa Y , Kurimasa A , Itamochi H , Harada T , Terakawa N , Masutani H *et al* (2008) Involvement of thioredoxin‐binding protein 2 in the antitumor activity of CD437. Cancer Sci 99, 2485–2490. 19018770 10.1111/j.1349-7006.2008.00979.x PMC11159347

[13] Chen Z , Lopez‐Ramos DA , Yoshihara E , Maeda Y , Masutani H , Sugie K , Maeda M and Yodoi J (2010) Thioredoxin‐binding protein‐2 (TBP‐2/VDUP1/TXNIP) regulates T‐cell sensitivity to glucocorticoid during HTLV‐I‐induced transformation. Leukemia 25, 440–448. 21151022 10.1038/leu.2010.286 PMC3072512

[14] Nishinaka Y , Nishiyama A , Masutani H , Oka S , Ahsan KM , Nakayama Y , Ishii Y , Nakamura H , Maeda M and Yodoi J (2004) Loss of thioredoxin‐binding protein‐2/vitamin D3 up‐regulated protein 1 in human T‐cell leukemia virus type I‐dependent T‐cell transformation: implications for adult T‐cell leukemia leukemogenesis. Cancer Res 64, 1287–1292. 14983878 10.1158/0008-5472.can-03-0908

[15] Yamaguchi F , Takata M , Kamitori K , Nonaka M , Dong Y , Sui L and Tokuda M (2008) Rare sugar D‐allose induces specific up‐regulation of TXNIP and subsequent G1 cell cycle arrest in hepatocellular carcinoma cells by stabilization of p27kip1. Int J Oncol 32, 377–385. 18202760

[16] Nishinaka Y , Masutani H , Oka S , Matsuo Y , Yamaguchi Y , Nishio K , Ishii Y and Yodoi J (2004) Importin alpha1 (Rch1) mediates nuclear translocation of thioredoxin‐binding protein‐2/vitamin D(3)‐ up‐regulated protein 1. J Biol Chem 279, 37559–37565. 15234975 10.1074/jbc.M405473200

[17] Sui L , Dong Y , Watanabe Y , Yamaguchi F , Hatano N , Tsukamoto I , Izumori K and Tokuda M (2005) The inhibitory effect and possible mechanisms of D‐allose on cancer cell proliferation. Int J Oncol 27, 907–912. 16142305

[18] Hirata Y , Saito M , Tsukamoto I , Yamaguchi F , Sui L , Kamitori K , Dong Y , Uehara E , Konishi R , Janjua N *et al* (2009) Analysis of the inhibitory mechanism of D‐allose on MOLT‐4F leukemia cell proliferation. J Biosci Bioeng 107, 562–583. 19393559 10.1016/j.jbiosc.2008.12.021

[19] Polyak K , Lee MH , Erdjument‐Bromage H , Koff A , Roberts JM , Tempst P and Massagué J (1994) Cloning of p27Kip1, a cyclin‐dependent kinase inhibitor and a potential mediator of extracellular antimitogenic signals. Cell 78, 59–66. 8033212 10.1016/0092-8674(94)90572-x

[20] Slingerland J and Pagano M (2000) Regulation of the Cdk inhibitor p27 and its deregulation in cancer. J Cell Physiol 183, 10–17. 10699961 10.1002/(SICI)1097-4652(200004)183:1<10::AID-JCP2>3.0.CO;2-I

[21] Tomoda K , Kubota Y and Kato J (1999) Degradation of the cyclin‐dependent‐kinase inhibitor p27^Kip1^ is instigated by Jab1. Nature 398, 160–165. 10086358 10.1038/18230

[22] Tomoda K , Kubota Y , Arata Y , Mori S , Maeda M , Tanaka T , Yoshida M , Yoneda‐Kato N and Kato JY (2002) The cytoplasmic shuttling and subsequent degradation of p27^Kip1^ mediated by Jab1/CSN5 and the COP9 signalosome complex. J Biol Chem 277, 2302–2310. 11704659 10.1074/jbc.M104431200

[23] Jeon JH , Lee KN , Hwang CY , Kwon KS , You KH and Choi I (2005) Tumor suppressor VDUP1 increases p27^kip1^ stability by inhibiting JAB1. Cancer Res 65, 4485–4489. 15930262 10.1158/0008-5472.CAN-04-2271

[24] Dephoure N , Zhou C , Villén J , Beausoleil SA , Bakalarski CE , Elledge SJ and Gygi SP (2008) A quantitative atlas of mitotic phosphorylation. Proc Natl Acad Sci USA 105, 10762–10767. 18669648 10.1073/pnas.0805139105 PMC2504835

[25] Patwari P , Chutkow WA , Cummings K , Verstraeten VLRM , Lammerding J , Schreiter ER and Lee RT (2009) Thioredoxin‐independent regulation of metabolism by the alpha‐arrestin proteins. J Biol Chem 284, 24996–25003. 19605364 10.1074/jbc.M109.018093 PMC2757204

[26] Cuenda A and Rousseau S (2007) p38 MAP‐kinases pathway regulation, function and role in human diseases. Biochem Biophys Acta 1773, 1358–1375. 17481747 10.1016/j.bbamcr.2007.03.010

[27] Sutter AP , Maaser K , Höpfner M , Huether A , Schuppan D and Scherübl H (2005) Cell cycle arrest and apoptosis induction in hepatocellular carcinoma cells by HMG‐CoA reductase inhibitors. Synergistic antiproliferative action with ligands of the peripheral benzodiazepine receptor. J Hepatol 43, 808–816. 16083991 10.1016/j.jhep.2005.04.010

[28] Schulze PC , Yoshioka J , Takahashi T , He Z , King GL and Lee RT (2004) Hyperglycemia promotes oxidative stress through inhibition of thioredoxin function by thioredoxin‐interacting protein. J Biol Chem 279, 30369–30374. 15128745 10.1074/jbc.M400549200

[29] Li X , Rong Y , Zhang M , Wang XL , LeMaire SA , Coselli JS , Zhang Y and Shen YH (2009) Up‐regulation of thioredoxin interacting protein (Txnip) by p38 MAPK and FOXO1 contributes to the impaired thioredoxin activity and increased ROS in glucose‐treated endothelial cells. Biochem Biophys Res Commun 381, 660–665. 19254690 10.1016/j.bbrc.2009.02.132 PMC2677205

[30] Ren Y , Shi Y , Wan Y , Li Y , Wu S , Li H , Zhang Y and Duan H (2010) p38 MAPK pathway is involved in high glucose‐induced thioredoxin interacting protein induction in mouse mesangial cells. FEBS Lett 584, 3480–3485. 20624390 10.1016/j.febslet.2010.07.010

[31] Fang S , Jin Y , Zheng H , Yan J , Cui Y , Bi H , Jia H , Zhang H , Wang Y , Na L *et al* (2011) High glucose condition upregulated Txnip expression level in rat mesangial cells through ROS/MEK/MAPK pathway. Mol Cell Biochem 347, 175–182. 20953987 10.1007/s11010-010-0626-z

[32] Alvarez CE (2008) On the origins of arrestin and rhodopsin. BMC Evol Biol 8, 222–234. 18664266 10.1186/1471-2148-8-222 PMC2515105

[33] Yamaguchi F , Kamitori K , Sanada K , Horii M , Dong Y , Sui L and Tokuda M (2008) Rare sugar D‐allose enhances anti‐tumor effect of 5‐fluorouracil on the human hepatocellular carcinoma cell line HuH‐7. J Biosci Bioeng 106, 248–252. 18930000 10.1263/jbb.106.248

[34] Hoshikawa H , Mori T and Mori N (2010) *In vitro* and *in vivo* effects of D‐allose: up‐regulation of thioredoxin‐interacting protein in head and neck cancer cells. Ann Otol Rhinol Laryngol 119, 567–571. 20860283 10.1177/000348941011900810

[35] Indo K , Hoshikawa H , Kamitori K , Yamaguchi F , Mori T , Tokuda M and Mori N (2014) Effects of D‐allose in combination with docetaxel in human head and neck cancer cells. Int J Oncol 45, 2044–2050. 25109398 10.3892/ijo.2014.2590

[36] Kanaji N , Kamitori K , Hossain A , Noguchi C , Katagi A , Kadowaki N and Tokuda M (2018) Additive antitumour effect of D‐allose in combination with cisplatin in non‐small cell lung cancer cells. Oncol Rep 39, 1292–1298. 29328484 10.3892/or.2018.6192

[37] Hoshikawa H , Kamitori K , Indo K , Mori T , Kamata M , Takahashi T and Tokuda M (2018) Combined treatment with D‐allose, docetaxel and radiation inhibits the tumor growth in an *in vivo* model of head and neck cancer. Oncol Lett 15, 3422–3428. 29456721 10.3892/ol.2018.7787 PMC5795844

